# In-Frame Indel Mutations in the Genome of the Blind Mexican Cavefish, *Astyanax mexicanus*

**DOI:** 10.1093/gbe/evz180

**Published:** 2019-08-19

**Authors:** Daniel Berning, Hannah Adams, Heidi Luc, Joshua B Gross

**Affiliations:** Department of Biological Sciences, University of Cincinnati

**Keywords:** RNA-sequencing, genetic lesion, *Astyanax*, troglomorphy

## Abstract

Organisms living in the subterranean biome evolve extreme characteristics including vision loss and sensory expansion. Despite prior work linking certain genes to Mendelian traits, the genetic basis for complex cave-associated traits remains unknown. Moreover, it is unclear if certain forms of genetic variation (e.g., indels, copy number variants) are more common in regressive evolution. Progress in this area has been limited by a lack of suitable natural model systems and genomic resources. In recent years, the Mexican tetra, *Astyanax mexicanus*, has advanced as a model for cave biology and regressive evolution. Here, we present the results of a genome-wide screen for in-frame indels using alignments of RNA-sequencing reads to the draft cavefish genome. Mutations were discovered in three genes associated with blood physiology (*mlf1*, *plg*, and *wdr1*), two genes associated with growth factor signaling (*ghrb*, *rnf126*), one gene linked to collagen defects (*mia3*), and one gene which may have a global epigenetic impact on gene expression (*mki67*). With one exception, polymorphisms were shared between Pachón and Tinaja cavefish lineages, and different from the surface-dwelling lineage. We confirmed the presence of mutations using direct Sanger sequencing and discovered remarkably similar developmental expression in both morphs despite substantial coding sequence alterations. Further, three mutated genes mapped near previously established quantitative trait loci associated with jaw size, condition factor, lens size, and neuromast variation. This work reveals previously unappreciated traits evolving in this species under environmental pressures (e.g., blood physiology) and provides insight to genetic changes underlying convergence of organisms evolving in complete darkness.

## Introduction

The blind Mexican tetra (*Astyanax mexicanus*) is a robust model for cave biology and evolution, having evolved extreme phenotypic features of troglomorphy including vision and pigmentation loss (Protas et al. 2008). This species includes an extant surface-dwelling form, to which distinct cave morphs may be interbred to produce experimental pedigrees. These pedigrees were employed in classical genetic studies to estimate the number of genes involved in trait losses (Wilkens 1988). In 2006, Protas et al. pioneered quantitative trait loci (QTL) analyses in this species using a recombination-based linkage map, and 8 years later the first cavefish genome draft was published (McGaugh et al. 2014). Despite the discovery of numerous genes and genetic regions associated with phenotypic differences, our understanding of the precise genetic mutations explaining key cave-associated traits remains limited (O’Quin and McGaugh 2015).

An emerging theme in evolutionary genetic studies of cavefish is the role of insertion/deletion (indel) coding mutations. Two Mendelian pigmentation phenotypes in cavefish have been linked to indels with clear functional consequences. Albinism is caused by genetic lesions impacting the *Oca2* locus, having evolved through different large-scale exonic deletions in two distinct cave localities (Protas et al. 2006). A second Mendelian phenotype, *brown*, is caused by a 2-bp deletion in the 5′ region of the open reading frame of the gene *Mc1r* which results in a truncated, nonfunctional receptor (Gross et al. 2009). In total, only three frameshift indel mutations are known from the *Astyanax* literature including two in the gene *Mc1r* (Gross et al. 2009, 2018), and two (in distinct populations) in the gene *Oca2* (Protas et al. 2006). These examples may represent rare circumstances of coding sequence mutations underlying simple, Mendelian phenotypes. Alternately, these examples may indicate a central role for indel mutations in the evolutionary loss of phenotypes.

Single nucleotide polymorphisms represent the most common form of genetic variation, with indels regarded as the second most abundant (Sergouniotis et al. 2016). Out-of-frame indels cause frameshifts to the mRNA coding sequence, which can lead to mRNA decay and an associated loss of protein expression (Guan et al. 2012). However, the impact of in-frame indels is more difficult to predict. The conventional wisdom is that the majority of these mutations have little impact on protein function. Recent studies, however, have shown that a 3-bp in-frame indel causes a loss-of-function *CFTR* allele in cystic fibrosis (Mullaney et al. 2010). Further, clinical and in silico modeling has revealed a role for in-frame indels in inherited forms of retinal dystrophy and childhood cataracts (Sergouniotis et al. 2016). Roughly 5% of genetic mutations impacting the tumor suppressor gene *AT-rich interactive domain 1A* (*ARID1A*) in gynecological cancers are in-frame indels, and functional analyses revealed these mutations had substantial impact on cellular proliferation and protein stability (Guan et al. 2012).

In this study, we analyzed the cavefish genome for in-frame indels to discover underappreciated alterations. Out-of-frame indels are more difficult to detect without comprehensive whole-genome sequencing, or targeted candidate gene studies. Indeed, one of the first *Astyanax* transcriptomic analyses (Hinaux et al. 2013) did not identify indel mutations in a set of vision-related genes. The comprehensive set of developmental transcriptomic sequencing we used here enabled a feasible approach to identifying this underexplored source of genetic variation. Using this data set, we sought to determine the frequency and potential impact of in-frame indels in the cavefish genome. Toward this end, we aligned a comprehensive set of Illumina reads, isolated across embryogenesis, to the draft cavefish genome. Using a system of stringent filtering, we identified sequence variants through comparisons of extant surface-dwelling morphs, and two geologically distinct cavefish populations (Pachón and Tinaja). Across the entire genome, we validated several genes harboring in-frame indel variants between cave and surface forms. Five of these genes were positioned near known QTL controlling five cave-associated phenotypes, and three of these genes were associated with blood physiology. This study presents the first genome-wide scan of high-throughput sequence variants in *Astyanax* validated through Sanger sequencing. Using this approach, we discovered diverse, mostly in-frame deletions in numerous genes, providing candidate genes for cave trait evolution. This work suggests that regressive evolution may not be accompanied by simple, widescale accumulation of indels. However, the technique we apply here revealed genetic mutations associated with unappreciated phenotypic alterations to blood physiology, growth factor signaling, cartilage development, and epigenetics.

## Materials and Methods

### Mutation Discovery and Expression Analyses

RNA-sequencing reads collected from four developmental stages (10, 24, 36, and 72 hours-post-fertlization [hpf]) of *Astyanax mexicanus* from the surface, Pachón and Tinaja populations were used in this study. All reads are available from the NCBI Sequence Reads Archive under BioProject ID PRJNA258661. This data set includes 12 experiments per population (36 total) inclusive of 4 stages, each sequenced in triplicate (see Stahl and Gross 2017). The Pachón cavefish genome (v.102.82) was used as a reference to which we aligned each population set of sequencing reads in a templated assembly (SeqMan NGEN v.15.0). To narrow the search for fixed variants, we filtered to identify only those genes harboring an indel relative to the reference genome. We analyzed scaffolds at least 1 Mb in size. To increase the likelihood of identifying *bona fide* mutations, we filtered the data sets to analyze only coding sequence regions with a depth of 50 or more aligned reads. After eliminating genes with <50 aligned reads, we then filtered based on percent genotype, yielding a set of genes with an indel present in 98–100% of the aligned reads. We note that this initial filtering was designed to maximize our ability to detect authentic indels. Although it was successful in identifying validated indel mutations (Results), it is likely that our depth of coverage threshold interfered with identification of genes undergoing nonsense-mediated decay, because these genes could demonstrate a depth of coverage well below our initial threshold.

We specifically searched for “indel” variants within our data set by comparison to the reference genome. To avoid false positive detection associated with misassembly at splicing junctions, we focused on mutations residing within exonic sequences (avoiding those near the boundary of exons). We then manually evaluated the resulting 88 putative hits (Pachón = 20, Surface = 39, and Tinaja = 29) to nominate the top candidates for subsequent polymerase chain reaction (PCR) validation. In the process, we identified several false positives which we attributed to splice variants because these were frequently found near the intron/exon boundaries. Expression values, based on RNA-sequencing studies, were obtained as normalized” reads per kilobase of transcript per million mapped reads (RPKM) values, based on previously published work (Stahl and Gross 2017). Expression was visualized for each gene using whole-mount in situ hybridization as described (Luc et al. 2019) using stage-matched 24-, 36-, and 72-hpf surface fish and cavefish embryos (from the Tinaja and Pachón populations).

### Sequence Analysis and Sanger Validation

To determine the degree of conservation of candidate genes, encoded protein sequences were aligned with MegAlign (v.13.0.2; DNAStar), using the ClustalV algorithm, to compare amino acid residues across multiple taxa. The reference and variant files for each gene were collated in SeqMan Pro (DNAStar) and the position of each indel was visualized using the “Strategy View” and “Conflicts” function. To test the robustness and accuracy of our discovered mutations using the DNAStar software suite, we performed a parallel analysis using a second sequence alignment program (CLC Genomics). We aligned the same reference and variant files and surveyed the consensus region harboring the putative indel mutation. In all but two instances, we identified precisely the same mutation in both software programs (supplementary fig. S1, Supplementary Material online). However, our results using DNAStar appear more reliable because all seven of the indel mutations we identified using this program were validated using traditional PCR amplification.

Flanking primers were designed to amplify the putative indel mutations in each candidate gene from genomic DNA. We used Primer3 (Rozen and Skaletsky 2000) to amplify a ∼600-bp fragment. The following primer pairs were used to validate indels: *ghrb*-forward (5′-AGGCGCACAGCTTTCTGATA-3′), *ghrb*-reverse (5′-CCGTCAACCATGGTATATTCTG-3′), *mia3*-forward (5′-GAGTCTACCGCAAACAGCAA-3′), *mia3*-reverse (5′-CATGCTGAAACTCACCAGGA-3′), *mki67*-forward (5′-AGGCTAAAACTCCGTCTCCA-3′), *mki67*-reverse (5′-GATTCTGCTTTTGCCTCTGG-3′), *mlf1*-forward (5′-GATGTGGGTGCTCAAAACAA-3′), *mlf1*-reverse (5′-CACAAAGTTTCGGTGCATGT-3′), *plg*-forward (5′-CTGCATAACAATACCAGCCAAC-3′), *plg*-reverse (5′-CAGCTCATAATCAGTTCGGTGT-3′), *rnf126*-forward (5′-AGTCCCGAACACACAACACA-3′), *rnf126*-reverse (5′-CGCCGTGTTTCTATTCCAGT-3′), *wdr1*-forward (5′-ACTGCACCACCTTCCTTGAG-3′), and *wdr1*-reverse (5′-GCCCTAGCCATTCCATGTT-3′). All primer pairs were tested using gDNA isolated from representative surface, Pachón, and Tinaja populations. To amplify each fragment, we performed routine PCR amplification (25 µl volume per sample) using a Bio-Rad C1000 Thermal Cycler with the following cycling conditions: 94 °C for 2 min; 94 °C for 30 s, 56 °C for 60 s, 72 °C for 60 s, cycled 35 times; and a final step of 72 °C for 10 min. Amplified DNA fragments were run on a 1.5% agarose gel with 10 µl of Gel Red Nucleic Acid Stain (Biotium) per 100 ml of agarose. Products of the correct estimated length for all three populations were isolated and purified using the QIAquick Gel Extraction Kit (Qiagen). Two samples of each purified fragment were subjected to direct Sanger sequencing using the forward and reverse primer (Operon). All sequences were aligned and analyzed using SeqMan Pro (DNAStar).

Each gene harboring an indel mutation was used to generate probe for visualizing gene expression with in situ hybridization using our published technique (see Luc et al. 2019). Images were taken with a Leica M205A stereomicroscope outfitted with a Leica DFC310FX camera and collected using LAS-X software suite and contrast adjusted using Adobe Photoshop CS3. Images of the left lateral side of developing embryos from 24, 36, and 72 hpf for each gene are presented in supplementary figure S2, Supplementary Material online.

To estimate which allelic form we discovered is most likely the ancestral type, we analyzed each sequence variant from surface fish, and both cavefish populations. We performed this analysis in two ways. First, using cavefish and surface fish alleles alongside the orthologous *Danio rerio* sequence, we directly compared sequence alignments using MegAlign v.15.3.0 (DNAStar). Second, we performed BLAST sequence analyses for all relevant alleles against the zebrafish genome and assessed the levels of sequence similarity. In all cases, surface fish alleles were more similar to the zebrafish sequences, when compared with the cavefish.

### QTL Position Analyses

Marker positions associated with published QTL were collated from prior studies (Protas et al. 2006, 2007, 2008; Gross et al. 2009, 2014; Yoshizawa et al. 2012; Kowalko et al. 2013; O’Quin et al. 2013) to identify phenotype, marker name, marker location, and scaffold number and position according to the draft *Astyanax* genome (ASTYANAX 2.0; NCBI). In the context of this positional information, we evaluated the positions of seven genes we identified bearing in-frame indel mutations. One gene, *plg*, was not placed to a chromosome in the current assembly. The remaining six were mapped according to their position relative to the genome (25 haploid chromosomes), and each individual chromosome upon which they reside. All data were collated and represented in a Circos plot (fig. 4), with genetic distances calculated between markers and genes based on the draft genome.

## Results and Discussion

### Seven Expressed Genes Demonstrate In-Frame Insertions/Deletions between Surface Fish and Two Different Cavefish Populations

Our analysis began with separate alignments of each morphotype (e.g., exclusive surface fish reads) to the Pachón genome reference (26,698 genes). We note that RNA-sequencing reads (Materials and Methods) may bias our discovery toward genes with high levels of expression during embryogenesis. This will likely lead to failure to identify frameshift-inducing indels, because they may otherwise be lost through nonsense-mediated decay and demonstrate low expression through RNA-sequencing approaches (Popp and Maquat 2016). Genes harboring destructive mutations are oftentimes expressed at lower levels, if at all, because resultant mRNA molecules can be targeted for degradation through nonsense-mediated decay. However, the genes causing albinism and *brown* (*Oca2* and *Mc1r*, respectively) have been detected through reverse transcription PCR (RT-PCR), in situ hybridization, and quantitative PCR studies (Stahl and Gross 2015), suggesting evolutionarily relevant pseudogenes can be expressed in *Astyanax*. Thus, although our filtering approaches identified authentic indel mutations, this approach introduced detection bias favoring highly expressed genes.

**Fig. 1.**
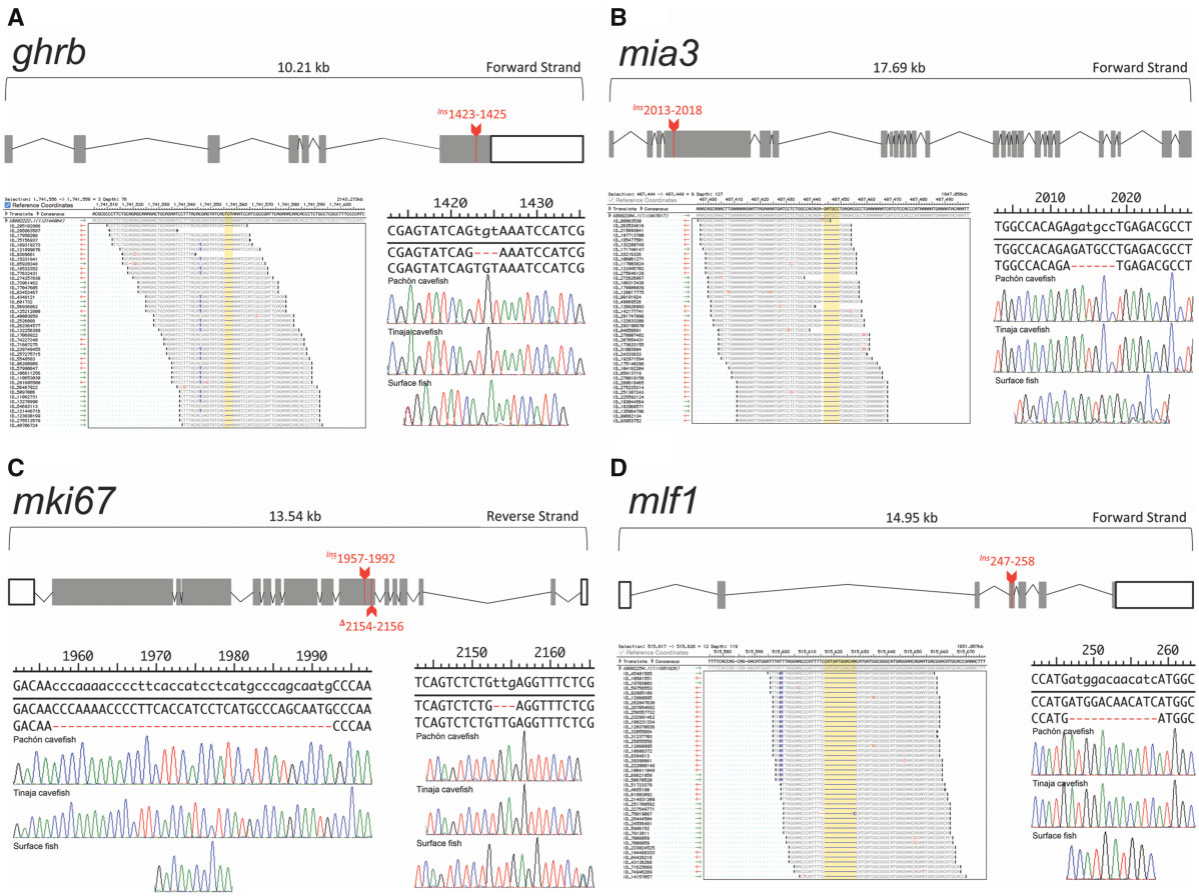
—RNA-sequencing alignments to the *Astyanax* genome uncovered indels in the expressed genes *ghrb*, *mia3*, *mki67*, and *mlf1*. The gene *ghrb* has a genomic length of ∼10.21 kb. Pachón and Tinaja cavefish harbor a 3-bp insertion in the seventh (last) exon of this gene, which was not present in surface fish (*A*). Similarly, the gene *mia3* (∼17.69 kb) demonstrates a 6-bp insertion (exon 4), which is only present in Pachón and Tinaja cavefish (*B*). The gene *mki67* (13.54 kb) has two sequential lesions in the ninth exon: a 36-bp insertion followed by a 3-bp deletion (*C*). Both mutations were observed in the cavefish lineages and were absent from surface fish. The gene *mlf1* (∼14.95 kb) demonstrates a 12-bp insertion in the third exon of Pachón and Tinaja cavefish, which was not present in surface fish (*D*). RNA-seq alignments are presented in (*A*), (*B*), and (*C*); detected lesions are highlighted in yellow. Validated lesions based on direct PCR sequencing are presented in (*A*)–(*D*) (Pachón top, Tinaja middle, and Surface bottom).

Alignment and filtering revealed several genes with nucleotide deletions divisible by a factor of 3. These indels are not anticipated to lead to frameshift mutations, but rather intact proteins. The first genetic lesion we discovered was a 3-bp insertion in the coding sequence of *growth hormone receptor b* (*ghrb*) in Pachón and Tinaja cavefish relative to surface fish. This mutation, *ghrb*^Ins1423^^–^^1425^ is an in-frame TGT insertion in the seventh exon (fig. 1*A* and table 1) in cavefish. The resultant mutation is predicted to impact the GHRB protein domain (table 2). Mutations in the human form of this gene are associated with Laron-type dwarfism, a rare autosomal recessive disorder refractory to growth hormone therapy (Godowski et al. 1989). Laron dwarfism is associated with low circulating levels of insulin-like growth factor (IGF). Interestingly, cavefish harbor genetic changes associated with better “body condition” (Protas et al. 2008), a surrogate for catabolic and anabolic metabolism. These metabolic changes may be linked to higher levels of circulating IGF (Peterson and Small 2004). However, recent studies have revealed that multiple populations harbor a sequence variant in the insulin receptor, which renders cave morphs resistant to insulin (Riddle et al. 2018).

**Table 1:** Summary of Indel Mutations Discovered through Alignment of RNA-Sequencing Reads to the Draft *Astyanax* Genome

Gene Name	Cave Fish Genome: Gene ID	Cave Fish Genome: Transcript ID	Insertion/Deletion	Population from Which Variant Was Identified	INDEL Location	Exon Position of Mutation (Total Number of Exons)	Gene cDNA Length (bp)	Genomic Orientation
*ghrb*	ENSAMXG00000009060	ENSAMXT00000009305.1	TGT insertion	Surface	*ghrb* ^Ins1423–1425^	7 (7)	1,650	Forward
*mia3*	ENSAMXG00000013444	ENSAMXT00000013833.1	GATGCC insertion	Surface	*mia3* ^Ins2013–2018^	4 (27)	5,667	Forward
*mki67*	ENSAMXG00000012117	ENSAMXT00000012463.1	CCCAAAACCCCTTCACCATCCTCATGCCCAGCAATG insertion and TTG deletion	Surface	*mki67* ^Ins1957–1992^ and *mki67*^Δ2154–2156^	9 (16)	6,750	Reverse
*mlf1*	ENSAMXG00000013404	ENSAMXT00000013791.1	ATGGACAACATC insertion	Surface	*mlf1* ^Ins247–258^	3 (7)	813	Forward
*plg*	ENSAMXG00000017876	ENSAMXT00000018418.1	T insertion and C deletion	Surface	*plg* ^Ins290^ and *plg*^Δ294^	4 (20)	2,451	Forward
*rnf126*	ENSAMXG00000012015	ENSAMXT00000012356.1	AGTTC deletion	Surface	*rnf126* ^Δ1046–1050^	11 (11)	1,063	Forward
*wdr1*	ENSAMXG00000003806	ENSAMXT00000003903.1	TGAGAT insertion	Tinaja	*wdr1^ins^* ^626–631^	6 (18)	1,674	Reverse

Note.—Surface form alleles are estimated to be the likely ancestral variant of each gene (see Materials and Methods). Therefore, each mutation is designated accordingly (e.g., an insertion in cavefish relative to the surface allele).

**Table 2:** Predicted Protein Domains Impacted in *Astyanax* Genes Harboring an Indel Mutation

Gene	Position of Mutation	Impacted Protein Domain(s)	Domain Interval
*ghrb*	*ghrb* ^Ins475^	GHBP	254–519
*mia3*	*mia3* ^Ins672–673^	None	N/A
*mki67*	*mki67* ^Ins656–667, Δ718^	Herpes_BLLF1, PTZ00449, PHA03247, retinal	477–718, 515–742, 203–676, 253–659
*mlf1*	*mlf1* ^Ins84–87^	Mlf1IP	81–210
*plg*	*plg* ^Ins97, Δ98^	PAN_AP_HGF, PAN_AP, PAN_1	32–105, 28–105, 31–105
*rnf126*	*rnf126* ^Δ349–350^	None	N/A
*wdr1*	*wdr1^ins^* ^209–210^	WD40 domain, WD40 repeat	54–390, 20–514

Note.—Surface form alleles are estimated to be the likely ancestral variant of each gene (see Materials and Methods). Therefore, each mutation is designated accordingly (e.g., an insertion in cavefish relative to the surface allele).

**Fig. 2.**
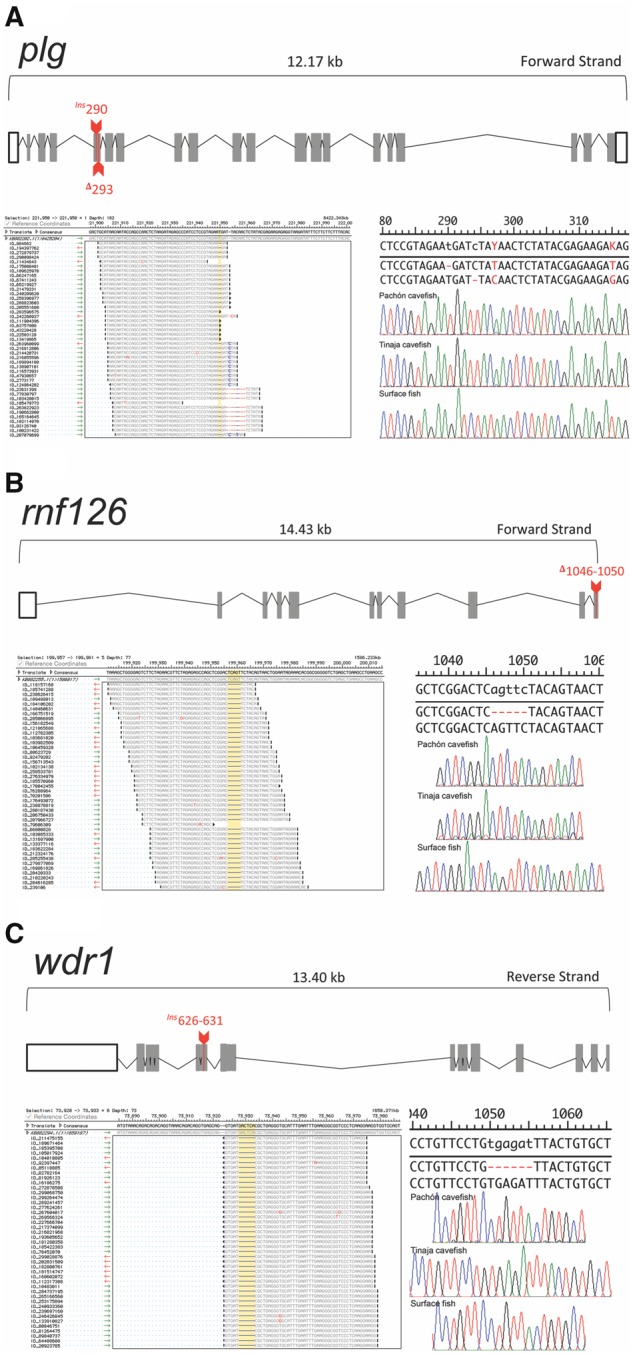
—RNA-sequencing alignments to the *Astyanax* genome uncovered indels in the expressed genes *plg*, *rnf126*, and *wdr1*. The gene *plg* has a genomic length of ∼12.17 kb. Cavefish from both localities harbor a 1-bp insertion followed by a subsequent 1-bp deletion in the fourth exon of this gene, neither of which is present in surface fish (*A*). The gene *rnf126* (∼14.43 kb) harbors a 5-bp deletion in the tenth exon, which is only present in the cavefish localities (*B*). The gene *wdr1* demonstrates a 6-bp insertion in Tinaja cavefish, which is absent from Pachón and surface-dwelling fish (*C*). RNA-seq alignments are presented in (*A*)–(*C*); detected lesions are highlighted in yellow. Validated lesions based on direct PCR sequencing are presented in (*A*)–(*C*) (Pachón top, Tinaja middle, and Surface bottom).

Interestingly, an Ecuadorian population of humans with mutations in the homologous gene (*GHR*) demonstrate an absence of diabetes as well as a significantly lower incidence of cancer (Guevara-Aguirre et al. 2011). This latter finding may impact age-dependent genomic stability, which in turn is associated with extended lifespan. Cave animals are known to exhibit dramatically longer lives (Venarsky et al. 2012), however the underlying mechanisms explaining this trait are elusive. We note, however, that the genetic lesion characterized in the Ecuadoran population is distinct from the lesion we identified in cavefish. The common Ecuadoran genotype causing Laron syndrome is a point mutation in exon 6 (an A-to-G splice-site mutation) leading to a protein lacking six amino acids in the extracellular domain, which likely results in a misfolded and degraded protein (Guevara-Aguirre et al. 2011). In contrast, the cavefish mutation resides near the terminus of the seventh exon and is an in-frame 3-bp insertion, rather than a splice-site mutation (fig. 1*A*). Functional analyses of ghrb in cavefish will determine if this mutation impacts longevity in cavefish through reduced or enhanced growth hormone receptor activity, and whether this mutation may have arisen through neutral loss as a secondary consequence of insulin resistance.

**Fig. 3.**
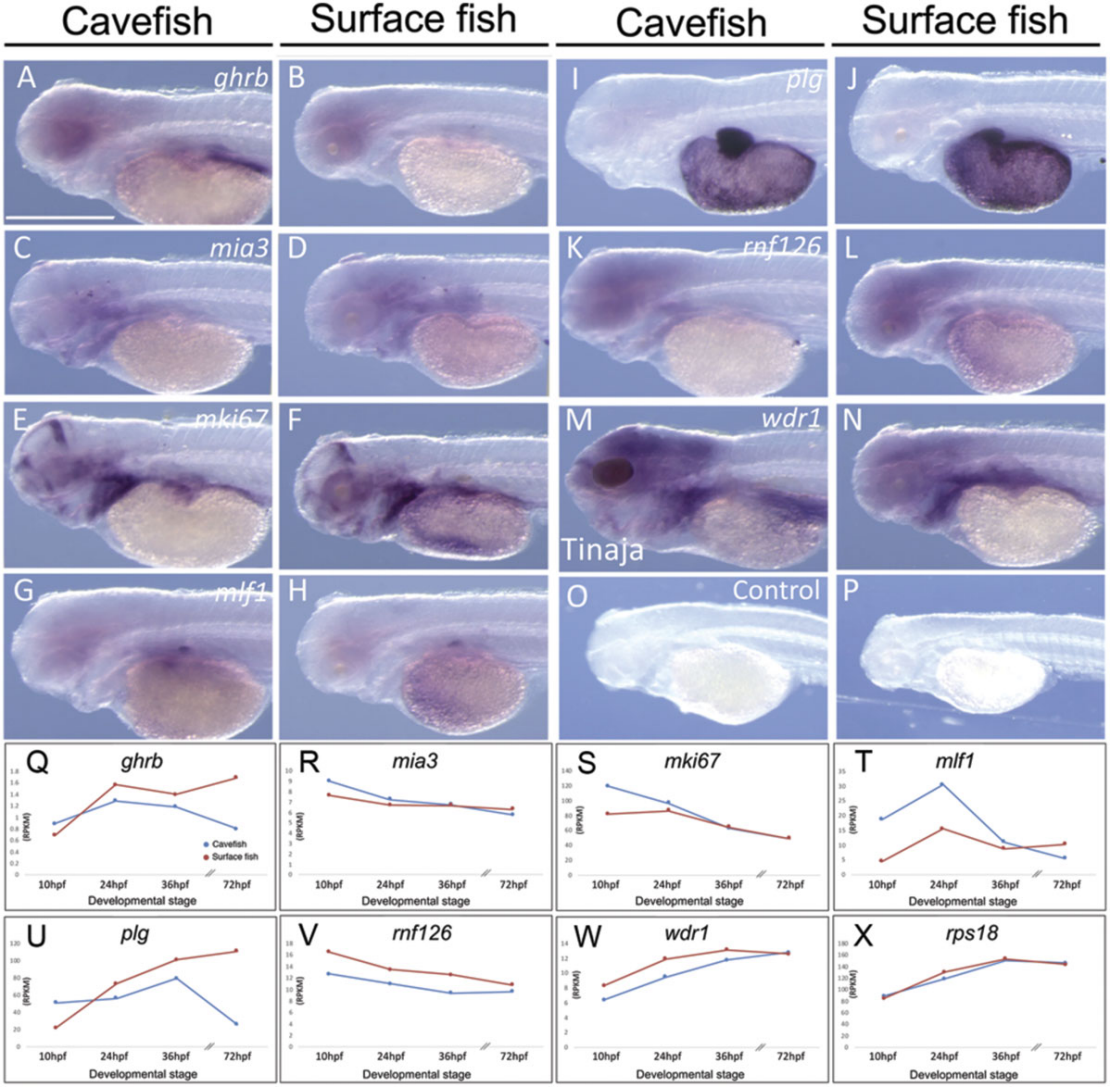
—Expression analyses reveal largely patterns between cave and surface fish embryos. Highly conserved patterns of gene expression based on whole-mount in situ hybridization were observed in 72 hpf embryos for the genes *ghrb* (*A*, *B*), *mia3* (*C*, *D*), *mki67* (*E*, *F*), *mlf1* (*G*, *H*), *plg* (*I*, *J*), *rnf126* (*K*, *L*), and *wdr1* (*M*, *N*). A no-probe control (*O*, *P*) is provided for comparison. RNA-sequencing expression data reveal largely conserved expression for genes across four stages of development (10, 24, 36, and 72 hpf), including *ghrb* (*Q*), *mia3* (*R*), and *mki67* (*S*). The gene *mlf1* (*T*) demonstrates differences in expression at the first two developmental stages, whereas *plg* (*U*) appears to be more conserved early in development, becoming polarized at the 72-hpf stage. Both *rnf126* (*V*) and *wdr1* (*W*) are highly similar in expression between morphs at all stages of development. For comparison, we present stable and highly similar expression patterns of *rps18* (*X*), a housekeeping gene used in prior transcriptomic studies. All whole-mount images were taken at 100×, except (*I*), (*J*), (*O*), and (*P*) which were taken at 45×. Scale bars = 500 µM.

Interestingly, we noted a slightly stronger pattern of expression for *ghrb* in the heads of cavefish embryos (fig. 3*A*). This expression may be associated with the larger jaws known from cave morphs compared with surface-dwellers (Retaux et al. 2008). We also noted a stronger pattern of expression for *ghrb* dorsal to the developing yolk sac of cavefish embryos (fig. 3*A*), however it is unclear if this expression is associated with differences in tissue development or a heterochronic shift in gene expression between morphs. RNA-seq analyses revealed that Pachón cavefish and surface fish gene expression is quite similar for the first three developmental stages assessed (10, 24, and 36 hpf), with surface fish expressing modestly higher levels than cavefish (fig. 3*Q*).

The second lesion we discovered was also an insertion in cavefish relative to surface fish in the gene *melanoma inhibitory activity protein 3* (*mia3*). This 6-base-pair insertion (GATGCC) is in the fourth of 27 exons (table 1) and is not predicted to cause a frameshift (fig. 1B). Further, this mutation, *mia3*^Ins2013^^–^^2018^, does not fall within an evolutionarily conserved region of the gene (table 2). Normally, this protein is associated with the endoplasmic reticulum and loading of collagen VII, and mice lacking this gene demonstrate global defects in cartilage maturation and bone mineralization (Wilson et al. 2011). Interestingly, similar to growth hormone receptor defects, *mia3*-null mice exhibit certain features of dwarfism (Malhotra and Erlmann 2011). Although unclear if the mutation we discovered impacts mia3 function, cavefish are known to demonstrate a number of skeletal defects, including intramembranous cranial bone fragmentation (Yamamoto et al. 2003) and the presence of supernumerary ribs (Dowling et al. 2002), which may stem in part from alterations in collagen deposition. Despite the presence of an indel in the gene *mia3*, expression comparison between cavefish (fig. 3*C*) and surface fish (fig. 3*D*) is nearly identical. Moreover, RNA-seq analysis revealed highly similar gene expression patterns across early development (fig. 3*R*).

In the gene *mki67*, we discovered two distinct mutations impacting the ninth of 16 exons (fig. 1C). The first lesion *mki67*^Ins1957^^–^^1992^ is a 36-base-pair insertion in both cavefish populations, relative to surface fish, resulting in the gain of 12 amino acid residues from positions 656 to 667 in the encoded protein (table 1). This mutation is anticipated to impact several overlapping functional protein domains, including the BLLF1, PTZ00449, PHA03247 and Retinal domains (table 2). The second genetic lesion, *mki67*^Δ2154^^–^^2156^ is a 3-base-pair deletion of TTG in cavefish, residing beyond a functional protein domain (table 2). The mki67 protein is essential for mitosis—maintaining chromosomes in the cytoplasm after nuclear envelope disassembly, which enables independent chromosomal motility (Cuylen et al. 2016). Our whole-mount expression analysis showed a complex pattern of *mki67* gene expression between cave (fig. 3*E*) and surface fish (fig. 3*F*), with the exception of absent staining in the developing eye vestiges of cavefish. Despite these differences, cavefish RNA-seq expression was higher at 10 hpf, but became nearly identical with surface fish from 24 to 72 hpf (fig. 3*S*). Although this protein is a marker of cell proliferation in mammalian cells, altering its levels does not substantially impact proliferation. Rather, it appears that Ki-67 plays a role in spatially organizing heterochromatin in proliferating cells, which are believed to have indirect effects on gene expression (Sobecki et al. 2016). Recent studies have begun to define a role for epigenetic regulation in the evolution of cave-associated traits. For instance, Rohner et al. (2013) demonstrated HSP90 inhibition can unmask substantial cryptic variation in natural cavefish; and Gore et al. (2018) demonstrated epigenetic silencing in cavefish via methylation-specific repression of vision-related genes. Therefore, if the mutation we discovered has a functional consequence, it may be through global changes in gene expression regulation that may have evolved because cavefish diverged from their above-ground ancestors.

The gene *myeloid leukemia factor 1* (*mlf1*) harbors a 12-base-pair insertion in cavefish relative to surface fish (fig. 1D), in the third of seven exons (table 1). This mutation, *mlf1*^Ins247^^–^^258^, results in the gain of four amino acid residues in the Mlf1IP domain of the resultant protein (table 2). This gene encodes an oncoprotein involved in hematopoietic cell determination and is associated with myelodysplastic syndrome (prior to development of acute myeloid leukemia; AML) in humans (Matsumoto et al. 2000). In their report, Matsumoto et al. (2000) demonstrated that elevated mlf levels are associated with de novo cases of AML. It is unknown if natural blind cavefish harbor alterations in blood cell determination, however *mlf1* represents one of three genes in which we identified a mutation putatively involved in blood physiology. During development, *mlf1* is expressed in a distinct spot within the flank dorsal to the developing yolk sac in both cave (fig. 3*G*) and surface (fig. 3*H*) morphs, which we assume is associated with the site of blood cell determination in the embryonic kidneys. Although RNA-sequencing shows divergent expression patterns early, by 36 hpf expression is similar in both morphs through ∼72 hpf (fig. 3*T*).

In the gene *plasminogen* (*plg*), we discovered two mutations separated by only four base pairs within the fourth exon (fig. 2A). This gene product is secreted in the blood and cleaved to generate plasmin (an enzyme that dissolves fibrin in blood clots). In null-*plg* mice, wound healing is substantially disrupted (Creemers et al. 2000), however it is unknown if this process is disrupted between surface- and cave-dwelling morphs. The first lesion we identified, *plg*^Ins290^, is a single base pair insertion at position 290 in surface fish which causes a frameshift. However, the second lesion, *plg*^Δ294^, restores the open reading frame at position 294 (table 1).

In humans, homozygous mutations in *plg* cause ligneous conjunctivitis—a rare disorder marked by the accumulation of fibrin-rich deposits on the underside of the eyelid (Schuster et al. 1997; Drew et al. 1998). It is intriguing to consider that structural alterations to plasminogen in cavefish may contribute, in part, to the presence of fibrous deposits in the empty orbital space of the cranium in adult cavefish (Wilkens 1988). Histological studies carried out several decades ago revealed a “rather fibrous” argentea in cavefish from the Pachón population (Wilkens 1971, 1988). The argentea is a part of the chorioid which covers the surface of the iris, conferring the silvery appearance in many teleost taxa (Kunz and Wise 1977). Our embryonic expression analysis showed the most distinct differences in expression for *plg*, which is expressed at very high levels in the developing liver of cavefish (fig. 3*I* and supplementary fig. S3, Supplementary Material online). Surface fish embryos demonstrate similar spatial patterns of expression, although expression is less strong in the liver at this developmental stage (fig. 3*J* and supplementary fig. S3, Supplementary Material online). Despite these apparent differences, RNA-seq studies showed cavefish have lower *plg* gene expression than surface fish by 72 hpf (fig. 3*U*).

The gene *ring finger protein 126* (*rnf126*) includes a five base pair deletion in cavefish in the eleventh exon (table 1). This mutation, *rnf126*^Δ1046^^–^^1050^, involves the deletion of AGTTC near the terminal end of the predicted open reading frame (fig. 2B). This mutation causes a frameshift, predicted to prematurely terminate the open reading frame compared with surface fish. This gene product is an E3 ubiquitin-protein ligase which is involved in proteasomal recycling of hydrophobic proteins in the cytoplasm (Krysztofinska et al. 2016). Prior work has shown that RNF126 and an associated protein (rabring7) play roles in ubiquitin-dependent sorting and downregulation of certain membrane receptors, such as epidermal growth factor receptor (Smith et al. 2013). Further, reduced expression of rnf126 is associated with stability of the insulin-like growth factor II receptor (*igf2r*) in cell culture assays (Huang et al. 2018). We observed the embryonic expression pattern of *rnf126* as diffuse and present only in the developing head of both morphotypes (fig. 3*K* and *L*). This pattern was congruent with RNA-seq studies, with modestly higher expression in surface fish (fig. 3*V*).

The gene *WD repeat-containing protein 1* (*wdr1*), harbored the only indel mutation unique to the Tinaja cave lineage. The variant we discovered, *wdr1^ins^*^626^^–^^631^, is an insertion of six bases (TGAGAT) in the sixth of 18 exons (table 1) of Tinaja cavefish. Interestingly, this sequence is one base pair different from a WD repeat (TGGGAT) and is nested within the WD40 repeat domain (fig. 2C; table 2). The *wdr1* gene product is involved in a number of critical cellular processes, including disassembly of actin filaments, cytokinesis, cell migration, and cardiomyocyte growth and maintenance (Yuan et al. 2014; Ono 2018). Interestingly, this gene is also implicated in the process of megakaryocyte maturation and suppresses platelet activity (Montenont et al. 2016)—two blood physiological processes. The gene *wdr1* appears to have a dorsally expanded expression in the head of Tinaja cavefish embryos (fig. 3*M*) compared with surface fish (fig. 3*N*), however we note that the overall expression pattern is diffuse and does not appear to be localized to specific developing tissues in cave or surface fish. Further, RNA-seq expression is virtually identical between Tinaja and surface fish embryos (fig. 3*W*) and is expressed at an especially low level across early development.

### Five Impacted Genes Colocalize to QTL Associated with Diverse Troglomorphic Features

**Fig. 4.**
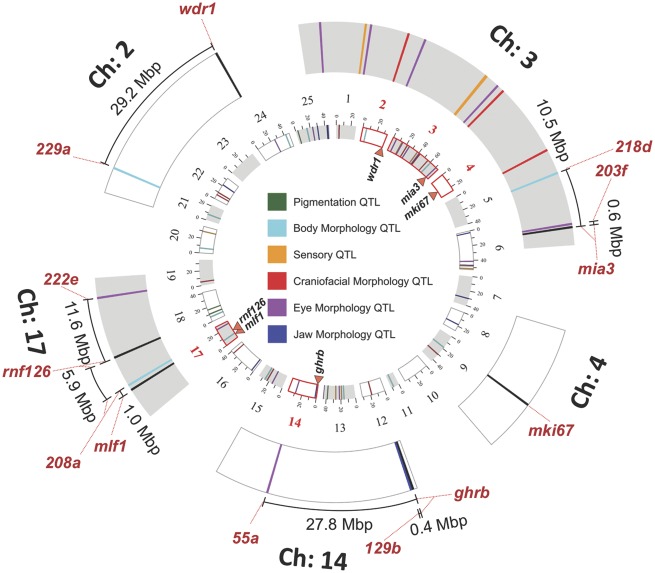
—A Circos representation of the relative positions of six genes with indel mutations and QTL associated with diverse traits in *Astyanax* cavefish. QTL positions are depicted relative to the draft *Astyanax* genome, alongside six candidate genes. On chromosome 2, the gene *wdr1* maps ∼29.2 Mb from microsatellite marker 229a (peak marker associated with anal fin ray morphology). The gene *mia3* resides on chromosome 3, near markers 203f (associated with lens size and superficial neuromasts) and 218d (associated with fin placement). The gene *ghrb* maps to chromosome 14, close to marker 129b (associated with jaw bone size), and more distantly to 55a (associated with eye size variation). The genes *mlf1* and *rnf126* both map to chromosome 17, near markers 208a (associated with condition factor), and 222e (associated with eye size, craniofacial bone size, and sex), respectively. Although *mki67* maps to chromosome 4, it is not located near any known QTL. In the current draft genome, *plg* resides on an unlocated chromosome.

In two previous cases, genes with indel mutations mapped to two simple Mendelian QTL (albinism and *brown*). We reasoned that the genes we discovered in this analysis may represent candidates for other traits with a genetic basis. Toward this end, we screened previously discovered peak loci associated with QTL studies in *Astyanax* published between 2006 and 2014 (reviewed and summarized in O’Quin and McGaugh [2015]). We evaluated traits according to six broad categories: pigmentation, body morphology, sensory, craniofacial morphology, eye morphology, and jaw morphology (fig. 4). DNA sequences associated with logarithm of the odds (LOD) peak markers for each QTL were localized to the *Astyanax* draft genome (2.0; NCBI), alongside positions for each gene with an indel mutation. We found a genomic location for 6 of the 7 genes harboring an indel (*plg* failed to map to a chromosome in the draft assembly), alongside genomic positions for 55 QTL from prior studies.

The gene *mki67* maps to chromosome 4, which does not include QTL from prior studies (fig. 4). However, the gene *wdr1* maps to chromosome 2 along with microsatellite marker 229a, which is associated with variation in anal fin ray morphology (Protas et al. 2008). This marker is quite distant (∼29.2 Mb) from *wdr1*, suggesting it may not play a role in the genetic control of this trait. The gene *mia3* is located on chromosome 3, very close to marker 203f (∼600 kb); and more distantly (∼10.5 Mb) to marker 218d (Protas et al. 2007). Marker 203f is associated with variation in lens size (Protas et al. 2007) and superficial neuromasts (Yoshizawa et al. 2012). Given the described roles for *mia3* in protein binding (GO:0005515) and lipoprotein transporter activity (GO:0042954), this gene product could conceivably play a role in the cellular/tissue organization associated with each these two complex phenotypes. At present, however, a role for *mia3* in these traits is unknown.

The gene *ghrb* maps to chromosome 14, ∼400 kb from the marker 129b, and more distantly to the marker 55a (27.8 Mb away). Interestingly, the closer marker (129b) is associated with adult jaw bone length (Protas et al. 2007), as well as condition factor (Protas et al. 2008). Administration of growth hormone in humans is associated with certain morphological changes, including larger adult jaw size. Although the phenotypic impact of the mutation we discovered in cavefish remains uncharacterized, it may contribute to the larger jaws known from natural cavefish compared with surface-dwelling forms (Retaux et al. 2008).

Two of the genes we discovered (*mlf1* and *rnf126*) reside on chromosome 17 in the *Astyanax* genome. The gene *mlf1* is located ∼1 Mb away from the microsatellite marker 208a, which is one of nine QTL associated with condition factor (Protas et al. 2008). It remains unclear if this gene plays a role in this trait, considering that mlf1 is associated with myeloid progenitor cell differentiation (GO:0002318). The gene *rnf126* similarly maps closest to marker 208a (∼5.9 Mb), and more distantly to marker 222e (∼11.6 Mb). This gene encodes an E3 ligase, which plays a role in localization of key proteins (e.g., epidermal growth factor receptor) and regulating retrograde sorting (e.g., mannose 6-phosphate receptor; Smith et al. 2013; Smith and McGlade 2014). The precise cellular basis for the “condition factor” phenotype is unknown; however, it is conceivable these genes impact directly (or indirectly) on this variable trait in *Astyanax*.

## Conclusions

In this study, we leveraged transcriptomic reads to identify several expressed in-frame indel mutations in the cavefish genome. This work revealed genes involved in diverse processes, including growth factor signaling, blood physiology, epigenetics, and collagen structure. Interestingly, expression analyses based on RNA-sequencing and whole-mount in situ hybridization suggest that, for the most part, gross expression-level changes have not accompanied the accumulation of these mutations. However, several genes mapped near previously established QTL, including a jaw size locus residing ∼400 kb from the gene *ghrb*, which is known to impact jaw structure in humans. This suggests that structural, rather than expression-level, changes may inform the role of these mutated genes in regressive evolution. Future studies focusing on functional validation of these putative mutations will be necessary for determining if these candidates represent the genetic basis for certain cave-associated phenotypes. Given the availability of large transcriptomic data sets in public repositories, similar genome-wide studies will serve as an important resource for nominating candidate genes underlying complex trait evolution, and improving our understanding of the impact of in-frame indel mutations. More specifically, this work identifies in-frame indels as a source of genetic variation that could reveal previously underappreciated traits evolving in natural model systems.

## Supplementary Material

Supplementary data are available at *Genome Biology and Evolution* online.

## Supplementary Material

evz180_Supplementary_Data

## Notes

**Data deposition:** NCBI SRA: BioProject ID PRJNA258661.
