# Supplementary material for: In-Frame Indel Mutations in the Genome of the Blind Mexican Cavefish, Astyanax mexicanus
Source: Genome Biol Evol. 2019 Aug 19;11(9):2563–73. doi: 10.1093/gbe/evz180 (PMC6751357; doi:10.1093/gbe/evz180)
Supplement: evz180_Supplementary_Data [file evz180_supplementary_data.zip › Legends suppl figs.docx]

**Supplemental Information**

Supplemental Figure S1. Parallel alignments performed using two different assembly programs yield near identical results. The results of sequence alignments that identified indel mutations is depicted for five genes. We identified precisely the same mutation in the gene ghrb using both DNAStar (top) and CLC Genomics assembly software (A). The same result was found for the gene *mia3* (B), as well as *mlf1* (C) and *plg* (D). For the gene rnf126, although a mutation was discovered using CLC Genomics, we believe the 5’ presence of a thymine and cytosine reflects an error of assembly, since these two bases are also present on the 3’ end of the mutation. Similarly, the mutation identified in the CLC alignment for *mlf1* (C) is shorter that the indel discovered using DNAStar (above). We believe that this is an artifact (i.e., a misassembly) attributed to the identity of the deleted sequence (CATGATGG) which is locally duplicated in this region. We interpret this misalignment to the improper placement of the deleted sequence in the CLC Genomics assembly. Since the seven indel mutations were validated using traditional PCR amplification, the DNAStar assembly was more accurate.

Supplemental Figure S2. Whole-mount *in situ* hybridization across development reveals embryological differences in expression between cave and surface fish. We performed whole-mount *in situ* hybridization across three developmental stages (24hpf, 36hpf, and 72hpf) for *ghrb* in cavefish (A–C) and surface fish (A’–C’). Similarly, we profile the developmental expression of *mia3* (D–F; D’–F’), *mki67* (G–I; G’–I’), *mlf1* (J–L; J’–L’), *plg* (M–O; M’–O’), *rnf126* (P–R; P’–R’), and *wdr1* (S–U; S’–U’). Unlabeled controls are depicted across the same developmental stages for cavefish (V–X) and surface fish (V’–X’). Scale bar = 500µM.

Supplemental Figure S3. Co-analysis of the liver marker *prox1* with *plg* confirms its early expression in the developing liver. Whole-mount in situ analysis of the gene prox1 reveals early expression over the dorsolateral yolk sac. This region of tissue is fated to give rise to the liver, the site of plasminogen production in the organism. In cavefish, *prox1* expression is absent at 24hpf (A), but becomes evident in rostral areas of the developing brain by 36hpf with a punctate region of expression in the dorsal yolk sac (arrowhead, B). By 72hpf, the region of expression associated with the liver is expanded (arrowhead, C). In surface fish, *prox1* expression is similar to cavefish, with little expression by 24hpf (G), two spots of expression in developing head by 36hpf (H), and more diffuse and less focal expression around the liver by 72hpf (I). In both cave and surface fish, *plg* is minimally expressed by 24hpf (D, J), and expression covers the entire yolk sac by 36hpf in cavefish (E) while it is expressed at lower levels at this stage in the ventral and posterior region of the yolk sac in surface fish (K). By 72hpf, *plg* is clearly being expressed in the developing liver in cavefish (arrowhead, F), and the lower level of expression in surface fish liver by 72hpf (L) reflects the similarly lower level of expression of *prox1* at this stage (I). Scale bar = 500µM.
