# Supplementary figures and images for: In-Frame Indel Mutations in the Genome of the Blind Mexican Cavefish, Astyanax mexicanus
Source: Genome Biol Evol. 2019 Aug 19;11(9):2563–73. doi: 10.1093/gbe/evz180 (PMC6751357; doi:10.1093/gbe/evz180)

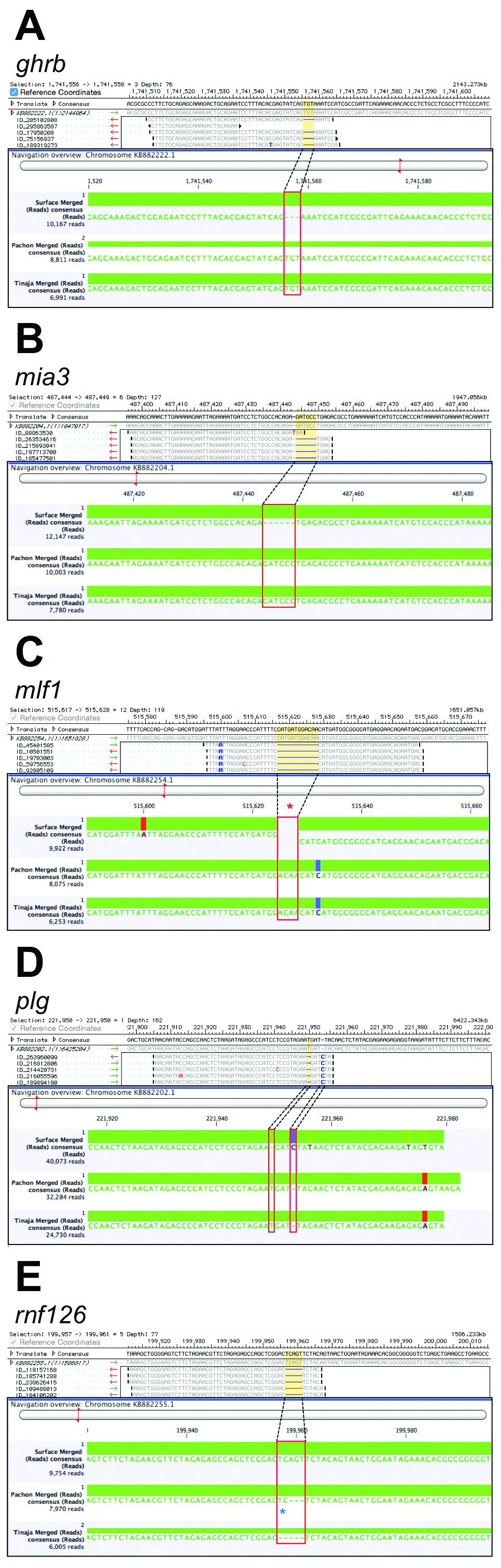

Supplement: evz180_Supplementary_Data [file evz180_supplementary_data.zip › Figure S1.tif]

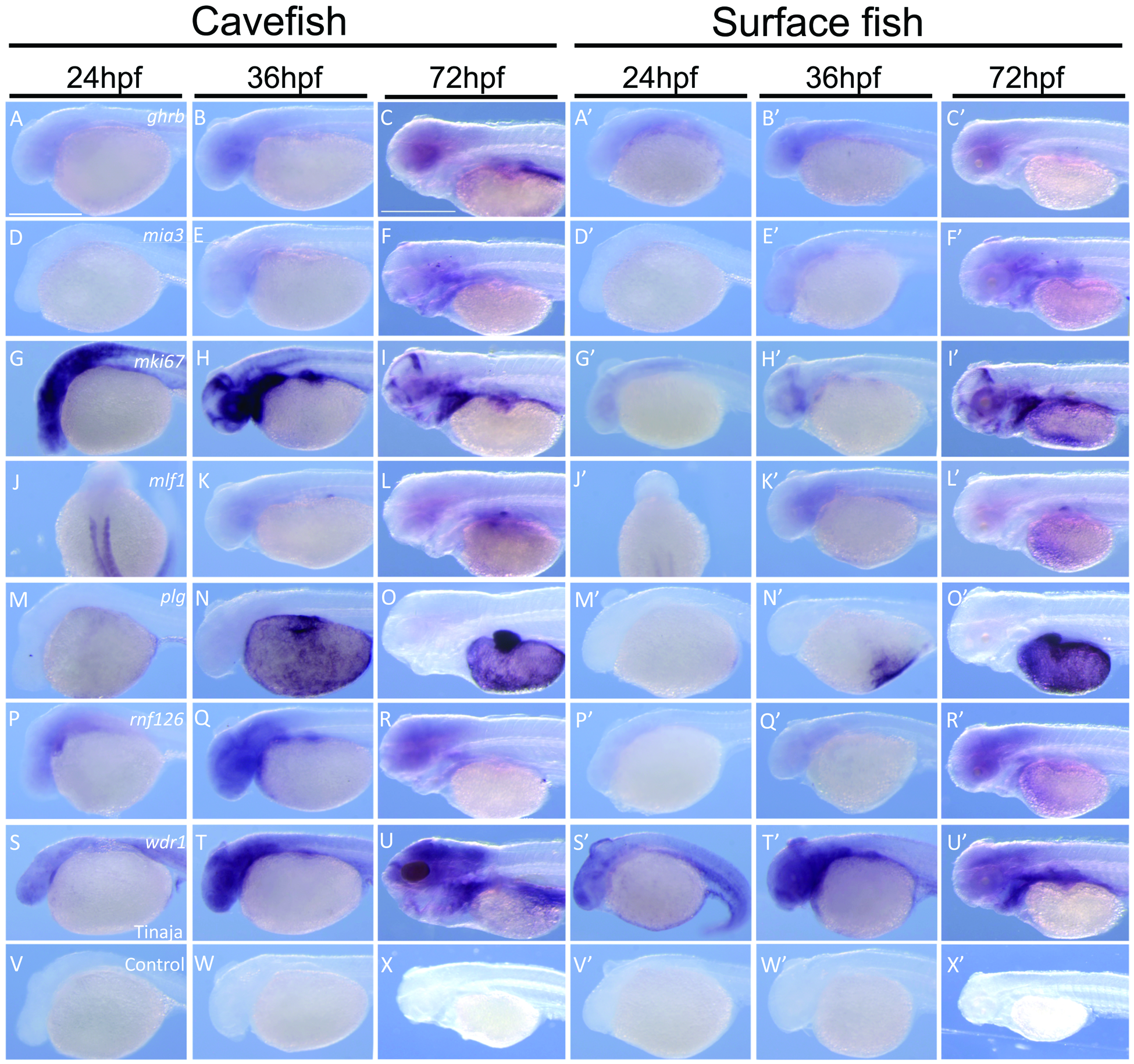

Supplement: evz180_Supplementary_Data [file evz180_supplementary_data.zip › Figure S2.tif]

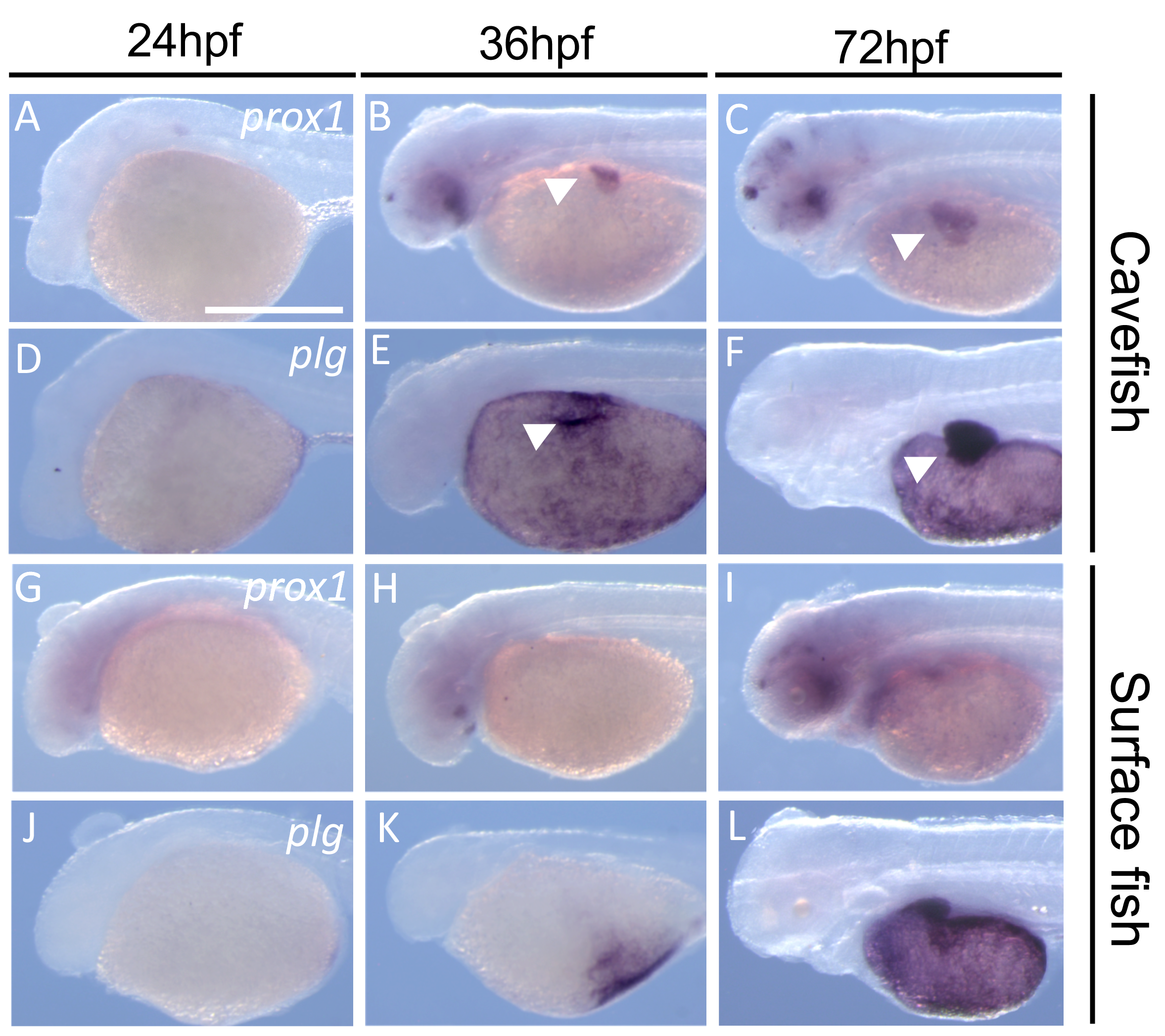

Supplement: evz180_Supplementary_Data [file evz180_supplementary_data.zip › Figure S3.tif]
